# Medical Knowledge Graph to Enhance Fraud, Waste, and Abuse Detection on Claim Data: Model Development and Performance Evaluation

**DOI:** 10.2196/17653

**Published:** 2020-07-23

**Authors:** Haixia Sun, Jin Xiao, Wei Zhu, Yilong He, Sheng Zhang, Xiaowei Xu, Li Hou, Jiao Li, Yuan Ni, Guotong Xie

**Affiliations:** 1 Institute of Medical Information & Library Chinese Academy of Medical Sciences Peking Union Medical College Beijing China; 2 PingAn Health Technology Shenzhen China

**Keywords:** medical knowledge graph, FWA detection

## Abstract

**Background:**

Fraud, Waste, and Abuse (FWA) detection is a significant yet challenging problem in the health insurance industry. An essential step in FWA detection is to check whether the medication is clinically reasonable with respect to the diagnosis. Currently, human experts with sufficient medical knowledge are required to perform this task. To reduce the cost, insurance inspectors tend to build an intelligent system to detect suspicious claims with inappropriate diagnoses/medications automatically.

**Objective:**

The aim of this study was to develop an automated method for making use of a medical knowledge graph to identify clinically suspected claims for FWA detection.

**Methods:**

First, we identified the medical knowledge that is required to assess the clinical rationality of the claims. We then searched for data sources that contain information to build such knowledge. In this study, we focused on Chinese medical knowledge. Second, we constructed a medical knowledge graph using unstructured knowledge. We used a deep learning–based method to extract the entities and relationships from the knowledge sources and developed a multilevel similarity matching approach to conduct the entity linking. To guarantee the quality of the medical knowledge graph, we involved human experts to review the entity and relationships with lower confidence. These reviewed results could be used to further improve the machine-learning models. Finally, we developed the rules to identify the suspected claims by reasoning according to the medical knowledge graph.

**Results:**

We collected 185,796 drug labels from the China Food and Drug Administration, 3390 types of disease information from medical textbooks (eg, symptoms, diagnosis, treatment, and prognosis), and information from 5272 examinations as the knowledge sources. The final medical knowledge graph includes 1,616,549 nodes and 5,963,444 edges. We designed three knowledge graph reasoning rules to identify three kinds of inappropriate diagnosis/medications. The experimental results showed that the medical knowledge graph helps to detect 70% of the suspected claims.

**Conclusions:**

The medical knowledge graph–based method successfully identified suspected cases of FWA (such as fraud diagnosis, excess prescription, and irrational prescription) from the claim documents, which helped to improve the efficiency of claim processing.

## Introduction

Currently, claim processing is a labor-intensive task for health insurance companies. For each claim document, the insurance inspector, who is usually a trained medical professional, needs to check whether the claim is reasonable from a clinical perspective, such as to catch any irrationality between a drug and diagnosis, or to check whether the examination is suitable for the diagnosis or symptoms. Detecting any signs of Fraud, Waste, and Abuse (FWA) is akin to looking for a needle in a haystack through claim data. The insurance company needs to hire people with sufficient medical knowledge, which significantly increases its human resource cost. Besides, claim processors still need to consult textbooks or the drug labels periodically as it is quite hard to remember details for all types of diseases, drugs, and examinations, which reduces the efficiency of claim processing. To improve the efficiency of the labor-intensive claim processing task, domain experts have devised some rules to generate a warning for suspected claims automatically. However, as the claims are coming from various hospitals that use different terminologies for drugs, examinations, and diagnoses, the coverage of fixed rules established by domain experts is relatively low. Moreover, as the drug information continues to be updated, the rules need to be updated correspondingly. To handle these challenges, knowledge graph technology could be used to represent unstructured medical knowledge such that the computer could perform reasoning on top of the knowledge graph to determine whether the claim is clinically reasonable automatically. Moreover, a method to build the knowledge graph automatically or with low human labor cost is indispensable.

Computational methods have been studied to detect FWA events [1-4]. However, it is difficult for these methods to collect comprehensive data supporting graph analysis results. Machine-learning methods failed to handle complex situations and provide interpretable evidence. There is also a gap between research and industry in FWA detection. Medical knowledge graph techniques provide a sound solution for interpretability. Recently, many medical knowledge graphs have been constructed based on medical terminology, ontology, clinical guidelines, medical encyclopedias, online forums, and electronic medical records [5-9]. For Chinese medical knowledge graph construction, natural language processing techniques have shown excellent performance on named entity recognition (NER; eg, disease, drug, and symptom) and relation extraction (eg, treatment, diagnosis) [5]. A challenge for medication information extraction from clinical notes is organization [10]. However, drug labels also contain valuable clinical information. A method that can extract high-accuracy information from drug labels is therefore expected. In addition, it is challenging to assess the effectiveness of a medical knowledge graph in an artificial intelligence app [9]. Disease-centered knowledge graphs [11,12] are tailored toward clinical decision-making support instead of using large-scale data without a curated graph. Similarly, a specific and curated medical knowledge graph is needed for enhancing FWA detection.

In this paper, we present a method to automatically build a medical knowledge graph for FWA detection in claim processing. To support FWA detection, a medical knowledge graph should cover the essential concepts such as diseases, drugs, examinations, symptoms, and the relationships between these concepts such as *<treat; drug, disease>*, *<interact; drug, drug>*, and *<check; disease, examination>*.

The main contributions of this study are as follows. First, we designed a medical knowledge graph schema for intelligent claim processing in health care insurance, and we collected recognized knowledge sources to support medical knowledge graph construction. Second, we built the medical knowledge graph using a deep learning–based method to extract entities and relationships from the knowledge sources automatically. We explored a human-machine collaboration to improve the quality of the medical knowledge graph. Finally, we applied the medical knowledge graph to empower claim processing in a health care insurance scenario.

## Methods

### Overview

**Figure 1:**
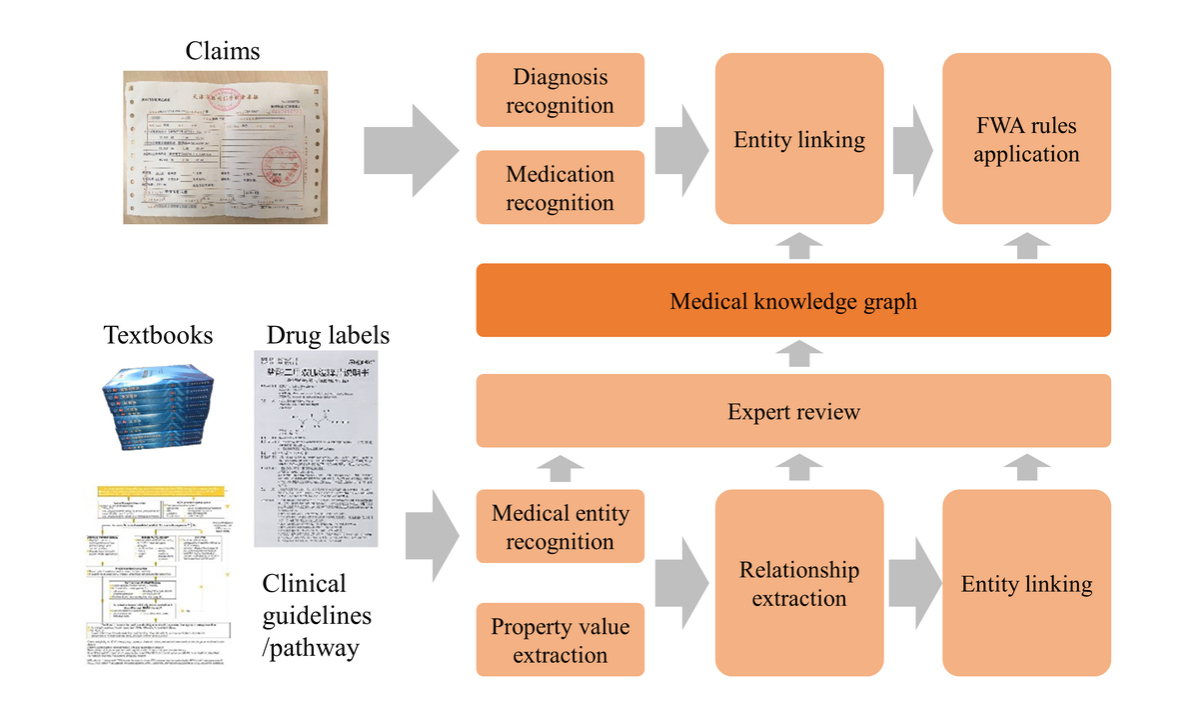
Overview of our methodology. FWA: Fraud, Waste and Abuse.

Figure 1 shows an overview of our methodology. We divided the method into an offline workflow and online workflow. The offline workflow conducts information extraction from various medical corpora to build a comprehensive medical knowledge graph. We further improved the knowledge graph quality through domain expert review. In the online step, given the claim documents, we first identified the diagnosis and medications from the claims and then linked the mentioned terms to our medical knowledge graph. Finally, we applied the FWA rules and knowledge graph reasoning to conduct an evaluation. In the following section, we will illustrate these steps in detail.

**Figure 2:**
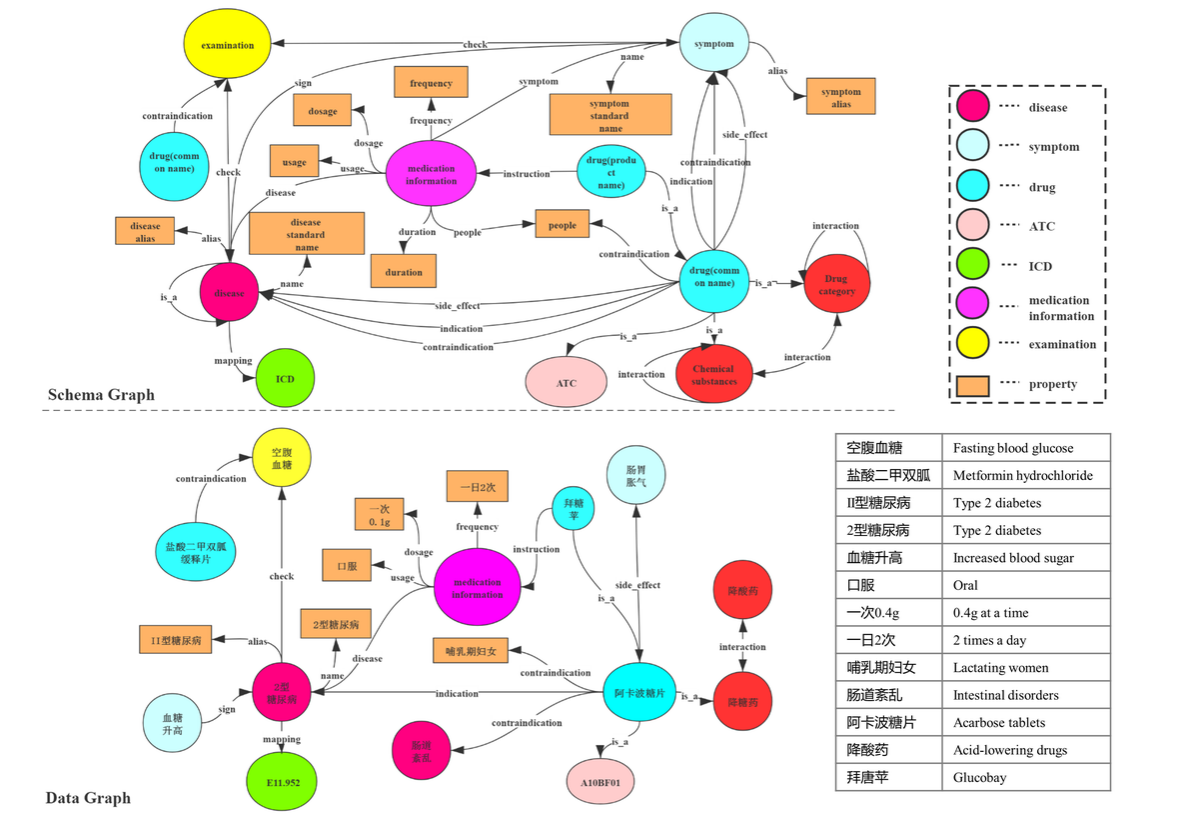
Medical knowledge graph schema (class) and a data graph example (instance). ATC: Anatomical Therapeutic Classification; ICD: International Classification of Diseases.

To build the knowledge graph, we first needed to define a knowledge graph schema (ie, establish concepts and relationships) according to the requirements in claim processing. Figure 2 shows the schema of the medical knowledge graph where the circles represent the concepts and the rectangles represent the data type property. We identified three kinds of essential concepts in the FWA scenario: disease, examination, and drug. For the disease concept, as the diagnosis in the claim documents uses the International Classification of Diseases (ICD)-10 [13] terminology, we also used this terminology in the knowledge graph. For the examination, we used the terminology for the service list of China social insurance. For the drugs, we considered the Anatomical Therapeutic Chemical (ATC) level name, the generic name, and the product name. Among these concepts, we identified seven types of beneficial relationships, as shown in Figure 2 (eg, *<interaction, drug, drug>*).

The above-required knowledge was collected from three sources: medical textbooks, drug labels, and clinical guidelines. We collected information on more than 3000 diseases and 1000 examinations from textbooks, 185,796 drug labels from the China Food and Drug Administration, and more than 2000 clinical guidelines from the Chinese Medical Association. In the following sections, we will introduce the algorithms used to identify the concepts and relationships from these sources.

### Named Entity Recognition

**Figure 3:**
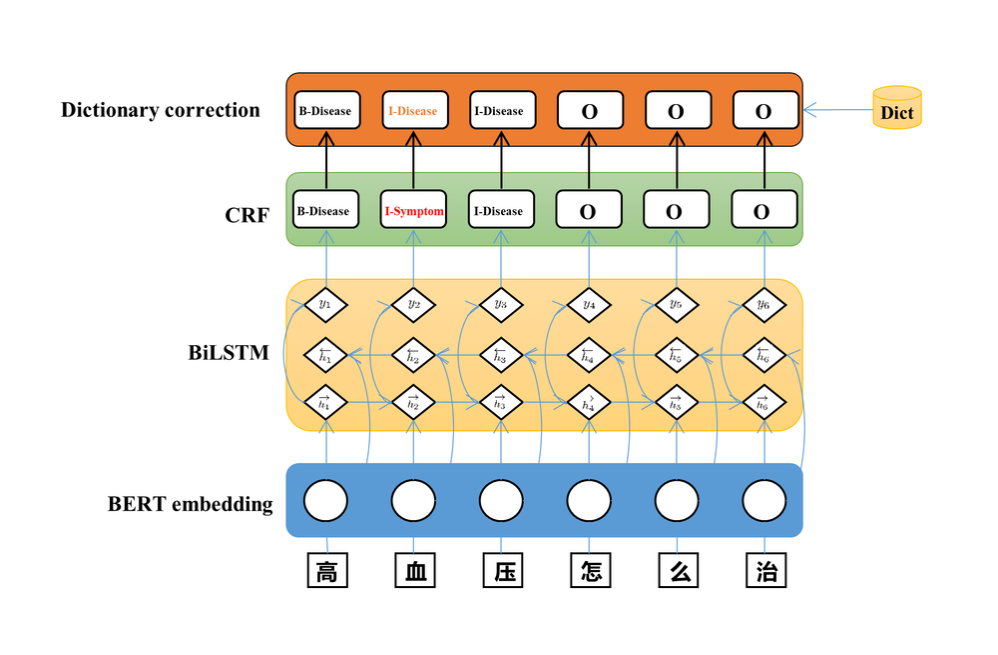
Structure of the hybrid system. BERT: Bidirectional Encoder Representations from Transformations; BiLSTM: bidirectional long short-term memory; CRF: conditional random field.

NER is used to detect medical entity mentions from unstructured data. As shown in Figure 3, we needed to identify five types of entities (ie, diseases, drugs, examinations, symptoms, and operation).

Although there are many Chinese NER methods [14-17], these methods still face many challenges, especially in the medical field. Therefore, we developed a hybrid method combining a neural network and dictionary-based system to optimize performance with limited training data, as shown in Figure 3. The input sentence first passes through the pretrained Bidirectional Encoder Representations from Transformations (BERT) model to obtain contextualized embeddings. Subsequently, there is a bidirectional Long Short-Term Memory-Conditional Random Field (BiLSTM-CRF) layer to provide preliminary predictions [18]. Finally, the predictions of the model would be corrected by a high-quality dictionary if any mistake is present. The description of the training data used is provided below.

#### Neural Network Model

Our model is an improved version based on conventional BiLSTM-CRF. We improved the model from the following aspects.

First, we replaced the tokenizer and word embedding with BERT [19], which is a Chinese-only pretrained language model (BERT Chinese-only model) provided by Google. By using such a pretrained language model, the effects of lacking training data can be alleviated since it provides more robust character and sentence representations.

Second, we used a feature engineering approach. We included many additional handcrafted features to the model. Neural networks have a good reputation for automatically capturing features. However, in the case of industrial application, handcrafted features can help to improve the robustness of the model. We extracted the following features. We used a word segmentation tool to extract the word segmentation soft boundary in which we used a Begin-Middle-End segmentation tag for each character of the text and the label was mapped to a low­dimensional vector by a randomly initialized matrix. Radical features were extracted, as Chinese characters are hieroglyphic, which means that the shape of each character can represent its actual meaning to some extent. In the medical domain, a character consisting of the radical “疒” is usually related to a disease or symptom. Another typical case is “月,” which is relevant to a body structure. In addition, we extracted the prefix/suffix feature. In Chinese, a word typically consists of more than one character, and some characters play the role of a prefix or suffix. For instance, a disease name often has the suffix “病” and drugs often have suffixes such as “胶囊” or “冲剂.”

#### Rule-Based Adjustment

Combining the predictions of a deep-learning NER model, manually developed rules, and dictionaries can be a difficult task. Results from the model and the dictionary can have conflicts, neither of which is always correct. After analyzing the results of the prediction of multiple experiments, we found that the most common mistakes that a model can make are inconsistent tagging, wrong entity type, and incomplete span. Inconsistent tagging means that a predicted entity instance is not tagged in the correct Beginning (B)-Inside (I)-Outside (O) format (eg, “I-­Disease I-­Disease”). A wrong entity type means the model gives out the wrong entity type. For example, it mistakes a disease for a drug, or it gives out an inconsistent entity type such as “B­-Disease I­-Drug I­-Disease” for a disease entity. Incomplete span, the most frequently detected problem, means that the model predicts the “O” label for a part of the entity instance. For example, the model outputs a tagging sequence “B-Disease I-Disease I-Disease O O O” for the original sequence “帕金森综合症” (Parkinson disease). The above three problems can also co-occur. Thus, after we obtained the model prediction, we conducted the following adjustments. First, we checked whether the span is complete by checking whether after adding the surrounding words, the entity span is in the dictionary. If so, the longer span is accepted; otherwise, the span is accepted as is. Second, if the entity is in the dictionary, then the entity type suggested by the dictionary is used; otherwise, the entity type given by the model is accepted. If the model gives inconsistent entity types such as “B­Disease I­Drug I­Disease,” the entity type that occurs more frequently in this entity instance is chosen (ie, “Disease” in this entity instance). Finally, the tags are adjusted following the B-I-O format.

### Property Value Extraction

The objective of property value extraction is to extract the property information corresponding to an entity from the unstructured text. In drug instructions, the properties we focused on mainly included the usage, frequency of administration, dosage, and treatment course, which are different when targeting different diseases or symptoms, or different populations [20,21].

**Figure 4:**
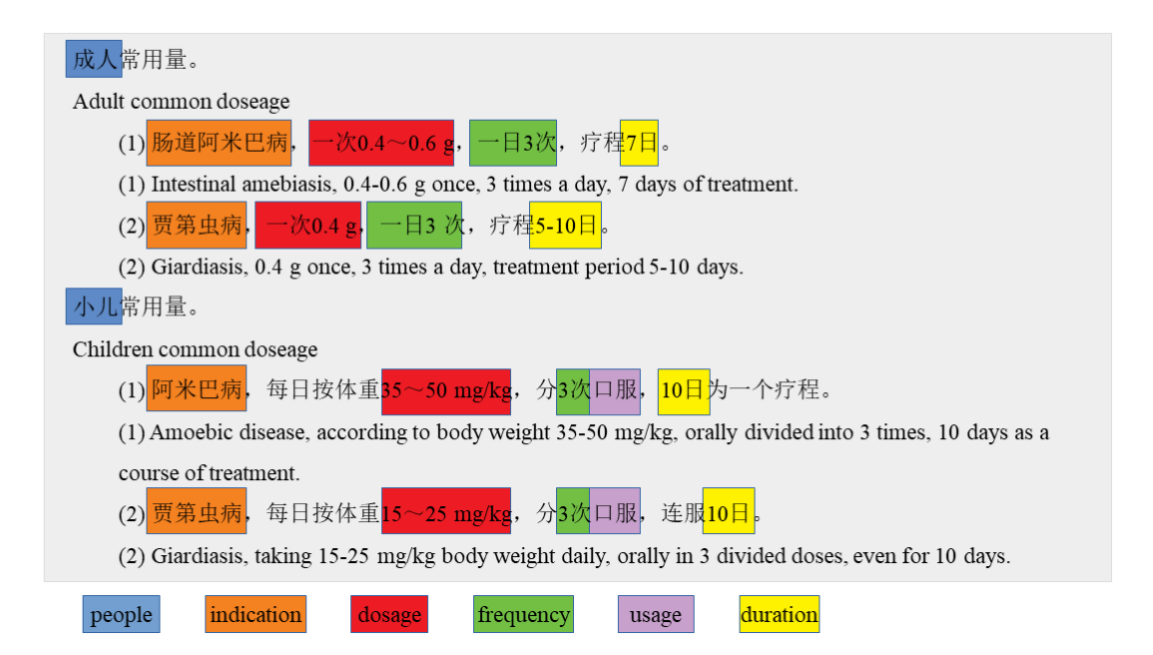
Illustration of the categories and values for properties that need to be extracted from usage section in the instruction of metronidazole tablets.

Figure 4 shows an interception of the usage fields in the instructions for the metronidazole tablet. The highlighted portion indicates the properties that need to be extracted. Table 1 shows the results of the two medication information entities. The property value of the field is mostly standardized, and it is easy to summarize the template. However, the main challenge we face is that for different populations with different diseases or symptoms, the detailed usage and dosage may be different.

**Table 1:** Property values extracted from the usage section in the instructions of metronidazole tablets.

Property	Entity 1	Entity 2	Entity 3	Entity 4
Usage	口服 (Oral)	口服 (Oral)	口服 (Oral)	口服 (Oral)
Dosage	0.4­0.6 g/次 (g/one time)	0.4g/次 (g/one time)	35­50 mg/kg	15­25 mg/kg
Frequency	一日 3 次 (3 times a day)	一日 3 次 (3 times a day)	3 次 (3 times a day)	3 次 (3 times a day)
Duration	7 日 (7 days)	5-10 日 (5-10 days)	10 日 (10 days)	10 日 (10 days)
Indication	肠道阿米巴病 (Intestinal amoebiasis)	贾第虫病 (Giardiasis)	阿米巴病 (Amoebiasis)	贾第虫病 (Giardiasis)
Population	成人 (Adults)	成人 (Adults)	儿童 (Children)	儿童 (Children)

To solve these problems, property value extraction for drug instructions is usually divided into two parts: property value recognition and property value combination. Property value recognition is used to locate boundaries and determine categories, and property value combination combines property values belonging to the same entity.

#### Property Value Recognition

Different property values require different methods. In addition to the model-based method for indication, the remaining property values are determined by the pattern-based method [22]. The following describes the extraction method of each property value.

##### Dosage, Frequency, Duration, Population

The properties of dose, frequency, duration, and population are similar in form and are a combination of numbers and units; thus, similar extraction methods can be used. Taking the dose as an example, the pattern is first used to extract all combinations of numbers and dosage units such as “gram” or “slice,” and then the context keywords are used to retain the combination so that context hit keywords form the property value of dosage. The population property value is considered since a description of the taboo property may exist, such as that the dosage is 2 times a day for children but prohibited for children under 1 year of age. Therefore, interference data should be filtered according to the context keyword (prohibited) instead of the reservation.

##### Usage

Usage refers to the administration method of a drug such as “口服” (oral). We developed a set of patterns, which are shown in Table 2, to extract usage property values. For drug instructions that do not specify usage, we built a mapping table to infer it according to the dosage form, as shown in Table 3.

**Table 2:** Patterns of property value extraction described by regular expression-like syntax.

Pattern	Description	Example
一[次日](num)+片	Dosage pattern; [num] refers to an integer or decimal	一次一粒 (One capsule at a time)
(num)+[片粒克…](/kg)	Dosage pattern for drugs by weight	10毫克/kg体重 (10 mg/kg body weight)
一日(0-9)+次	Frequency pattern for the frequency of medication by day	一日3次 (t.i.d^a^)
疗程(0-9)+[日天]	Duration pattern	连续10天 (10 consecutive days)
(0-9)+岁(以[上下])?	Population pattern for age	18岁以下患者 (Patients under the age of 18)
[口嚼吞泡饮]服	Usage pattern for oral drugs	饭后口服 (Orally after meals)
静脉.{0,2}注射	Usage pattern for intravenous injection	静脉内注射 (intravenous injection) 140 mg/ml

^a^t.i.d: ter in die (3 times a day).

**Table 3:** Mapping table of the dosage form to usage.

Dosage form	Usage
缓释片(sustained release tablets)	口服 (oral)
肠溶片 (enteric-coated tablets)	口服 (oral)
直肠栓剂 (rectal suppositories)	肛门用药 (anal medication)
贴剂 (patch)	外用 (external)
洗剂 (lotion)	外用 (external)

##### Indication

An “indication” for a drug refers to the use of that drug for treating a disease. For example, diabetes is an indication for insulin. Following the previous section, we employed a BERT-BiLSTM-CRF model with manually designed features for indication detection, and the detected entities were linked to our medical knowledge graph.

#### Property Combination

The property values of the same medication information entity need to be combined. In most cases, all types of property values for the same entity will appear in the same sentence. For a small number of the remaining cases, we aggregated the extracted information via three heuristic rules based on linguistic patterns in the drug instructions as follows: (1) usage, dosage, and duration usually appear at the end; (2) if a property value does not appear in the description of the current entity but appears in the previous entity, the property value is usually the same as the previous entity; (3) if the population changes, the disease, frequency, and duration will also change.

Figure 4 and Table 1 illustrate the above three rules. Following rule (1), the duration (eg, “10日为一个疗程”, 10 days as a treatment course) appears last in the sentence. For entity 4, the sentence mentioning it does not specify the population group. However, following rule (2), we know that the population should be children, according to entity 3. All properties of entity 2 and entity 3 are different because of the change of population, as indicated by rule (3).

Therefore, most of the property values in the same sentence can be directly combined. Otherwise, we manually combined the extracted information to ensure precision and improve performance.

### Relation Extraction

**Figure 5:**
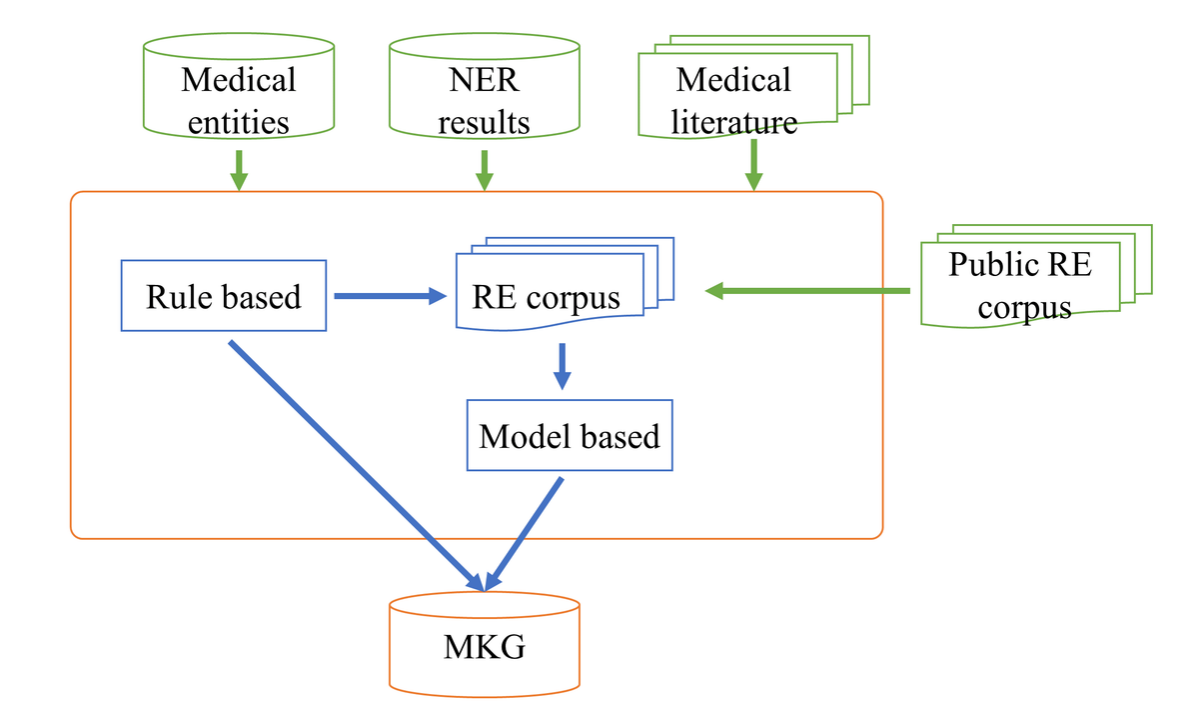
Framework of medical relation extraction (RE). MKG: medical knowledge graph; NER: named entity recognition.

Medical relation extraction refers to the semantic relationship between medical entities defined in the medical knowledge graph schema [23]. The main types of medical relations considered in this study include drug-drug interactions (DDIs), indications, and contraindications. Detailed information of the dataset is discussed further below. The medical relationship extraction framework proposed in this paper mainly includes two parts: a distant supervision method and a model-based method, as shown in Figure 5. In the distant supervision method, the medical relation extraction templates are formulated based on part­of­speech, syntactic structure, specific keywords, and expert medical knowledge [24]. The precision of medical relationships extracted by rule-based methods is high, but the recall is low. The amount of relations acquired by the rule-based method depends on the quantity and quality of the templates. In the model-based method, deep-learning models, especially those employing an attention mechanism, automatically contextualize the entity pairs together with their context information. Thus, they can generalize well and improve the recall of relation extraction [25].

#### Rule-Based Relation Extraction

We use the DDI relation as an example to demonstrate how to use rules for relation extraction. The relationship between drugs defined in the schema of the medical knowledge graph is divided into three categories: promotion, contraindication, and none (no relationship). Promotion indicates that two drugs can promote the efficacy of each other, contraindication means that two drugs will cause adverse reactions when taken at the same time, and none is no interaction between the two drugs. Some representative examples and patterns are provided in Table 4 and Table 5. The patterns were summarized manually after reading a small portion of the data. After the text data passes through an NER model, the entity instances are replaced with entity type symbols (eg, “吲哚美辛与胰岛素一起使用可以加强降糖效果,” indomethacin, when used with insulin, can enhance the hypoglycemic effect, becomes “[Drug]与[Drug]一起使用可以加强降糖效果,” [Drug], when used with [Drug], can enhance the hypoglycemic effect), and the patterns can identify the relation between the two entities.

**Table 4:** Categories of drug-drug interaction relations.

Categories	Explanation	Example
Promotion	Promote the efficacy of each other	吲哚美辛与胰岛素一起使用，可以加强降糖效果 (Indomethacin, when used with insulin, can enhance the hypoglycemic effect)
Contraindication	Produce adverse reactions when taken at the same time	吲哚美辛与秋水仙碱合用时可增加长胃溃疡及出血的危险 (Indomethacin combined with colchicine can increase the risk of long stomach ulcers and bleeding)
None	No interaction between the two drugs	阿德福韦酯和拉米夫定合用，两种药物的药代动力学特征都不改变 (When adefovir dipivoxil and lamivudine are used together, the pharmacokinetic characteristics of both drugs remained unchanged)

**Table 5:** Patterns for extracting drug-drug interaction relations.

Pattern	Description	Example
[…]禁止[…]合用	Contraindication; the sentence contains “禁忌” and “合用”	硝苯地平禁止与利福平合用 (Nifedipine is prohibited to be used with rifampicin)
[…]和[…]配伍禁忌	Contraindication; the sentence contains “配伍禁忌”	氯甲苯酸与青霉素有配伍禁忌 (Chlorotoluenic acid is contraindicated with penicillin)
[…]干扰[…]作用	Contraindication; the sentence contains “干扰” and “作用”	磺胺嘧啶片有可能干扰青霉素类药物的杀菌作用 (Sulfadiazine tablets may interfere with the bactericidal action of penicillin drugs)
[…]加强[…]效果	Promotion; the sentence contains “加强” and “效果”	盐酸昂丹司琼口腔崩解片与地塞米松合用可加强止吐效果 (Ondansetron hydrochloride orally disintegrating tablets combined with dexamethasone can enhance the antiemetic effect)
[…]增强[…]疗效	Promotion; the sentence contains “增强” and “疗效”	维生素C可增强盐酸吗啉胍注射液的疗效 (Vitamin C enhances the efficacy of morpholine hydrochloride injection)
[…]与[…]无相互作用	None; the sentence contains “无相互作用”	扎来普隆胶囊与帕罗西丁无相互作用 (Zaleplon capsules have no interaction with paroxetine)
[…]不改变[…]作用	None; the sentence contains “不改变” and “作用”	苯磺酸氨氯地平片不改变华法林的凝血酶原作用时间 (Amlodipine besylate tablets did not change the prothrombin time of warfarin)

#### Model-Based Relation Extraction

We experimented with a series of models for our relation extraction tasks, including piecewise convolutional neural networks (PCNN) [26], BiLSTM [18], and PCNN with adversarial training (PCNN+AT) [27]. Finally, a comparison was made among the models.

**Figure 6:**
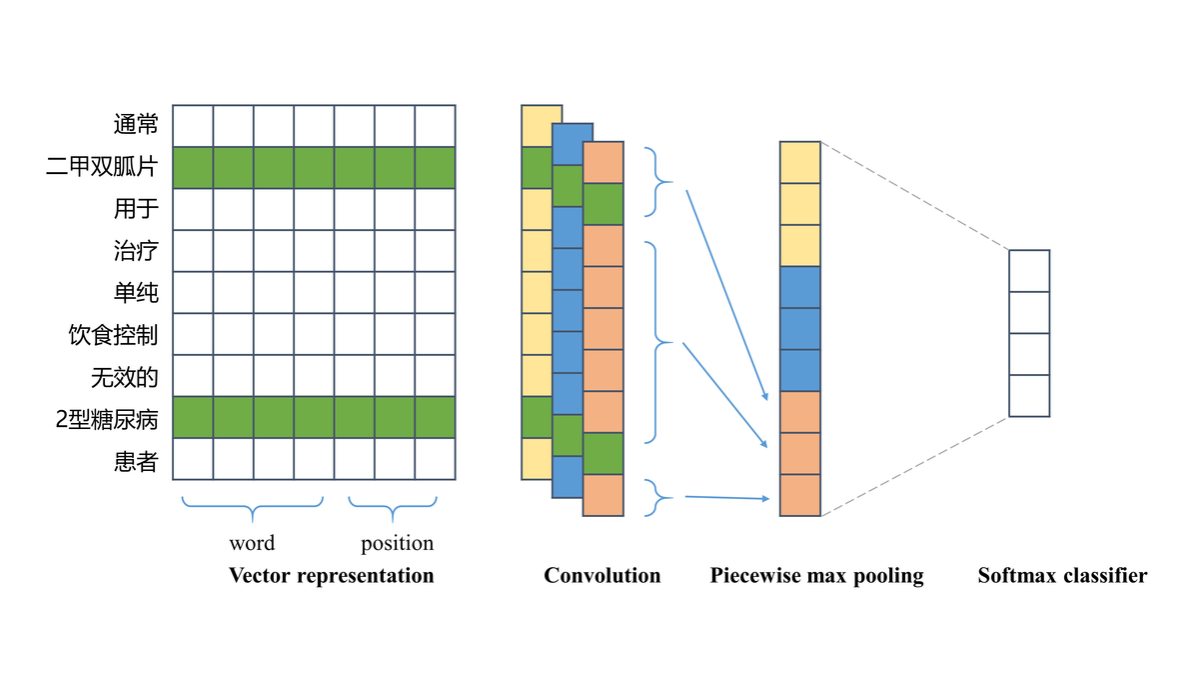
Piecewise convolutional neural networks architecture.

**Figure 7:**
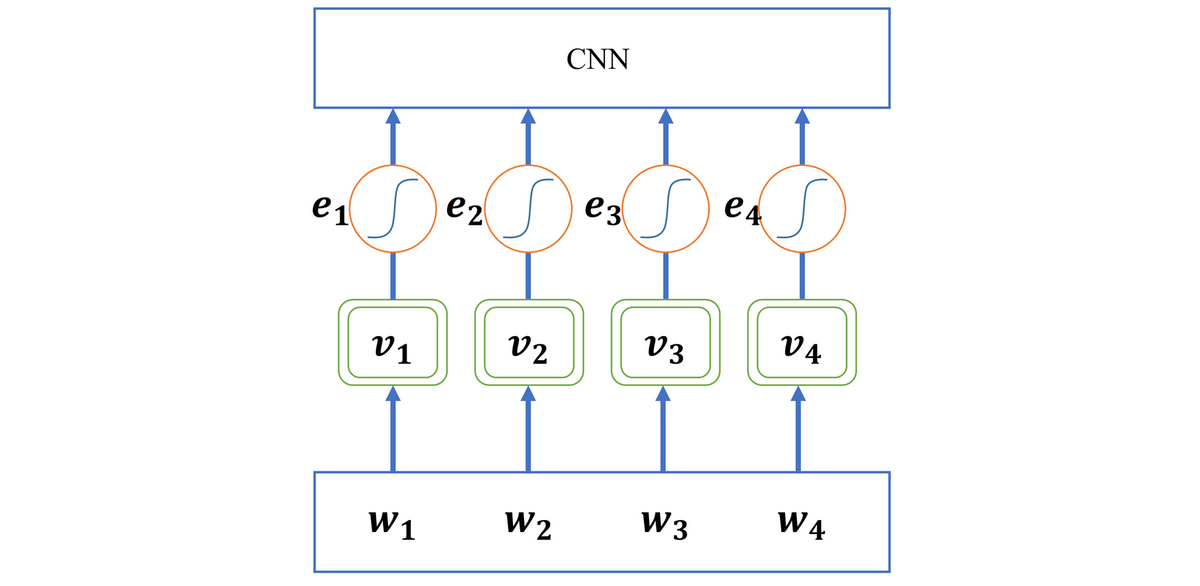
Computation graph of encoding a sentence xi with adversarial training. ei denotes the adversarial perturbation xi. Dropout is placed on the output of the variables in the double-lined rectangles. CNN: convolutional neural network.

Figure 6 depicts the PCNN model [26]. The sentence is first transformed into vectors. A convolution kernel is then applied, followed by a piecewise max pooling operation. Finally, the pooled features are sent to a softmax classifier to predict the relationship between two entities. To further improve the robustness of the model, we applied AT to improve the robustness of classifiers to small worst-case perturbations by calculating the gradient direction of loss function to the data. Since AT generates continuous disturbances, we added antagonistic noise at the word-embedding level. The network is shown in Figure 7.

### Knowledge Graph Fusion

Knowledge graph fusion can be regarded as an ontology alignment task in our workflow, which has been studied extensively in the literature [28-30]. In this paper, we present the task with unique domain characteristics in the medical field on fusing knowledge cards.

The previous entity extraction step would introduce the entity mentions that are unknown terms in the existing medical knowledge graph. In this case, one must decide whether an entity mention is a variant of some term in the medical knowledge graph or a new entity, which requires a precise entity normalization system. To build such a system, three difficulties are encountered. First, typos or discrepancies in transliterations may occur in online documents; for example, “唐尿病” is a frequent typo of “糖尿病” (diabetes), akin to diabites (misspelling) and diabetes, and “咪康唑” (miconazole) and “密康唑” (miconazole) are both transliterations of miconazole. Second, some entity mentions look quite alike, but represent quite distinct entities; for example, “ii 型糖尿病” (type 2 diabetes) and “i 型糖尿病” (type 1 diabetes) are quite similar, but they are very different entities. Third, there can be some discrepancies in expressing the same component of an entity name; for example, “手部擦伤” (hand abrasion) can be easily expressed as “手擦伤” (hand abrasion) since “手部” and “手” both mean hand.

The above three difficulties make it nontrivial to build a term canonicalization system, and previous systems have not addressed the above issues altogether [31-33]. One might consider combining more sophisticated machine-learning models such as neural networks along with the lexicon features and edit distances. However, sophisticated machine-learning models are challenging to train with limited labeled data and their results are not explainable.

**Figure 8:**
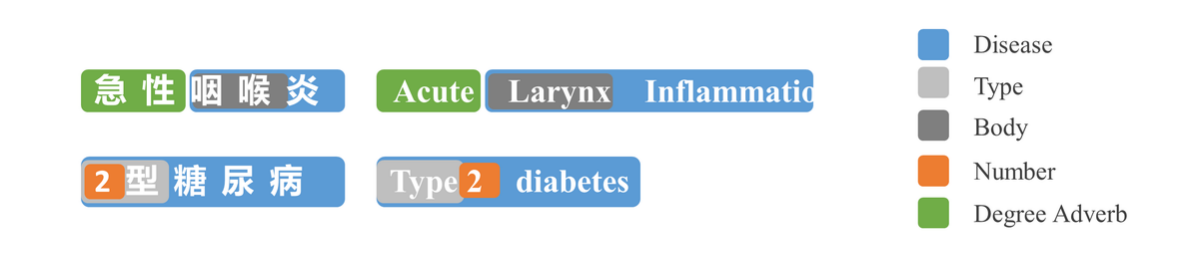
Sample of semantic unit’s category in disease.

To effectively address the above difficulties, we designed a multilevel fine-grained similarity score system. First, on the whole, we built a multilevel string matching algorithm, which we call ZhFuzzyED, considering three levels (token, radical, and pronunciation edit distance) so that the similarity score is less sensitive to typos and transliteration differences. Second, diving deeper into the components of entity names, we found that an entity mention such as a disease entity mention usually consists of semantic units such as body structure, negation, degree adverb, some adjective describing the type or stage, and the core term that defines the disease (as shown in Figure 8). Based on this observation, we collected and categorized 11 groups of semantic units. For each semantic unit category, a subgraph can be built to measure the similarity score between two semantic units. For example, “手臂” (arm) and “前肢” (forelimb) are similar, although their surface forms are different. Similarity scores concerning different semantic unit categories are weighted along with the string level similarity score.

**Figure 9:**
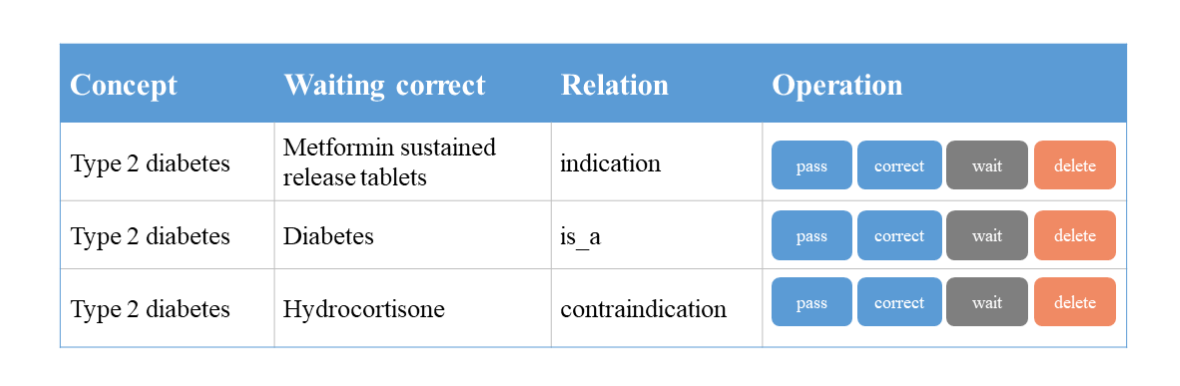
Design of the knowledge correction system. We examined the information extracted automatically, corrected the errors, and included the information in the medical knowledge graph, which was made available for the next round of information extraction and downstream tasks.

Natural language processing in the medical field is complicated and challenging. Thus, models and algorithms sometimes fail, and manual verification is essential. Therefore, we developed a tool (web app) to enable human-machine cooperation for knowledge graph fusion and knowledge graph quality control. The main design of the app is shown in Figure 9.

In this step, when a new entity mention comes in, we first search in the medical knowledge graph (usually via an invert index) for a possible matched entity and then the candidates are reranked via the above similarity score system. If the best-scored candidate still obtains a low score, the entity mention is considered to be an unknown entity, waiting to be added or corrected manually by experts. Otherwise, it is considered a term for best-scored known entity. This process is equivalent to a cycle of a self­learning process since the new terms added to the medical knowledge graph can improve the accuracy of our workflow at the next round of iteration.

### Applying the Medical Knowledge Graph for Claim Processing

In this section, we discuss how to use the medical knowledge graph to conduct automatic FWA detection in claim processing. Given a claim document, in the first step, we need to identify the diagnosis, examinations, and medications in the claims.

As the medical entities in a claim are extracted by optical character recognition or from various hospital information systems, these terms may follow different terminologies and may contain errors. Thus, term normalization is the foundation. We first used the aforementioned multilevel string matching algorithm (ZhFuzzyED) to perform term normalization.

After the entity mentions in a claim were linked to entities in the medical knowledge graph, we checked the following three suspicious scenarios.

#### Fraud Diagnosis

Fraud diagnosis is suspected when the disease does not match the indication of treatment. In this condition, the relation between a drug and disease can be used for detecting the mismatch case. There are three types of scenarios: (1) a drug does not have the disease as an indication; (2) the disease is a contraindication of the drug; and (3) no suitable drugs for treating the disease appear in this claim.

#### Excess Prescription

Excess prescription refers to excessive medical care such as one disease corresponding to many drugs in a claim, which is not medically necessary.

#### Irrational Prescription

Drugs prescribed in a claim have interactions. If the drugs in a visit record have interactions, especially when the interaction is harmful, the claim is considered to be fraudulent.

Inferring new facts from existing knowledge graphs is a form of an explainable reasoning process. The above scenarios could not be directly queried from the medical knowledge graph. Therefore, further reasoning on queries is required. Multihop knowledge graph reasoning was applied for our FWA detection. The graph reasoning rules are shown in Table 6.

**Figure 10:**
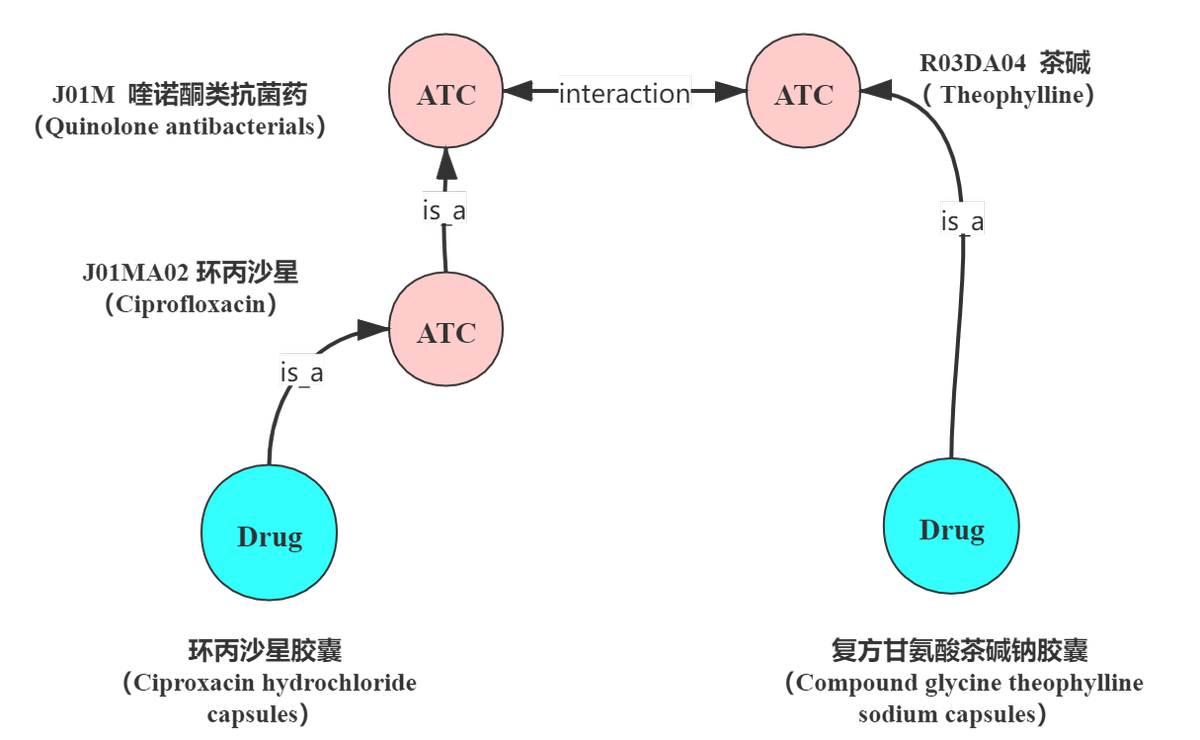
Example of graph query and graph reasoning. ATC: Anatomical Therapeutic Classification.

For example, as shown in Figure 10, the drug interaction relations are applied on the ATC level, whereas the relations are usually derived from the level of the common drug name (generic chemical name of a drug) extracted from the drug instructions. The occurrence of drug interactions is usually due to the chemical composition of a drug, which is the ATC code. Thus, to check whether two drugs have an interaction can provide an extension of query on ATC concepts.

**Table 6:** Graph reasoning processing in Fraud, Waste, and Abuse detection.

Suspicious scenarios	Graph reasoning rule (Cypher-like syntax)
Fraud diagnosis	(Disease)-[:is_a]-(Disease)<-[:indication]-(Drug)-[:is_a*0..1]->(ATC^a^)<-[:is_a*0..1]-(Drug)
Excess prescription	(Drug)-[:is_a]->(ATC)<-[:is_a]-(Drug)-[:indication]->(Disease)-[:is_a]-(Disease)
Irrational prescription	(Drug)-[:is_a]->(ATC)-[:is_a*0..3]->(ATC)<-[:is_a*0..3]-(ATC)<-[:is_a]-(Drug)

^a^ATC: Anatomical Therapeutic Classification.

## Results

### Datasets for Model Training

Our NER corpus was drawn from drug descriptions, encyclopedia pages for medical entities, and the literature so that the model trained can adapt to different scenarios. We prioritized documents that are related to entities that are common or medically important, which were split into 10,889 sentences. The annotation process followed the majority voting rule; that is, if two annotators did not agree on the annotation of the same sentence, then a senior annotator, who is a more experienced medical practitioner, made the final annotation. To save labor costs, our annotation is carried out in an active learning fashion as introduced by Chen et al [34]. For example, we first annotated the first 500 sentences using a medical dictionary, and then annotated them fully. Following the uncertainty-based sampling method, a pool of 1500 sentences was sampled. After the 2000 sentences were annotated, a better model could be obtained on the larger dataset. After repeating this step for a few iterations, we obtained our annotated dataset with less labor and higher quality in the sense that the models trained on it will perform better than a random sampled dataset.

The relation extraction dataset was built on the same corpus. The preannotation takes advantage of the technique of distant supervision in addition to active learning [35]. Distant supervision means that if two entities in a sentence are both in the medical knowledge graph, we assume that their relation in the sentence is in agreement with their relation in the medical knowledge graph. In the active learning procedure, if distant supervision detects relations in a sentence, we will prioritize on annotating this sentence. Annotators are responsible for determining that the distant supervised relation instance is correct, and whether there are other relation instances in the sentence. The whole annotation procedure gives out 21,657 relation instances, and the labor cost is estimated to be reduced by 4.3 times due to distant supervision and active learning.

### Model Performances

#### Performance of Named Entity Recognition

The annotated dataset was split into 8:2 training:test datasets. We compared three kinds of NER models: the deep-learning model only, the model with hand-crafted features, and the model with hand-crafted features and manually designed rules. Detailed results on the test set are shown in Table 7, demonstrating that the hand-crafted features are effective for performance improvement. In addition, the designed rules could further improve the performance of NER significantly.

**Table 7:** Performance of the named entity recognition models based on the entity level F1 value.

Variable	Model only	Model+feature	Model+feature +rules
Disease	0.901	0.921	*0.924* ^a^
Symptom	0.792	0.793	*0.801*
Examination	0.742	*0.744*	0.742
Drug	0.798	0.806	*0.821*
Operation	0.763	0.772	*0.784*
Overall	0.833	0.841	*0.850*

^a^Values in italics indicate the best performance on the same dataset.

#### Relation Extraction

The annotated dataset was split into 8:2 training:test sets. For relation extraction, we conducted experiments to evaluate the effectiveness of the three models and report the performances on the test set in Table 8. The PCNN and PCNN+AT models were described in the Methods section. The convolutional neural network (CNN) model is simply the PCNN model with vanilla pooling instead of piecewise pooling. We observed that the piecewise pooling is import for adequately representing the features of a sentence in the relation extraction task. Moreover, the PCNN+AT model had the best performance since it is more robust.

**Table 8:** Results of each model for overall relation extraction.

Model	Precision	Recall	F1 score
PCNN^a^	0.68	0.80	0.73
CNN^b^	0.55	0.76	0.64
PCNN+AT^c^	*0.75* ^d^	*0.84*	*0.80*

^a^PCNN: piecewise convolutional neural network.

^b^CNN: convolutional neural networks

^c^AT: adversarial training.

^d^Values in italics indicate the best performance.

### Statistics of the Medical Knowledge Graph

**Figure 11:**
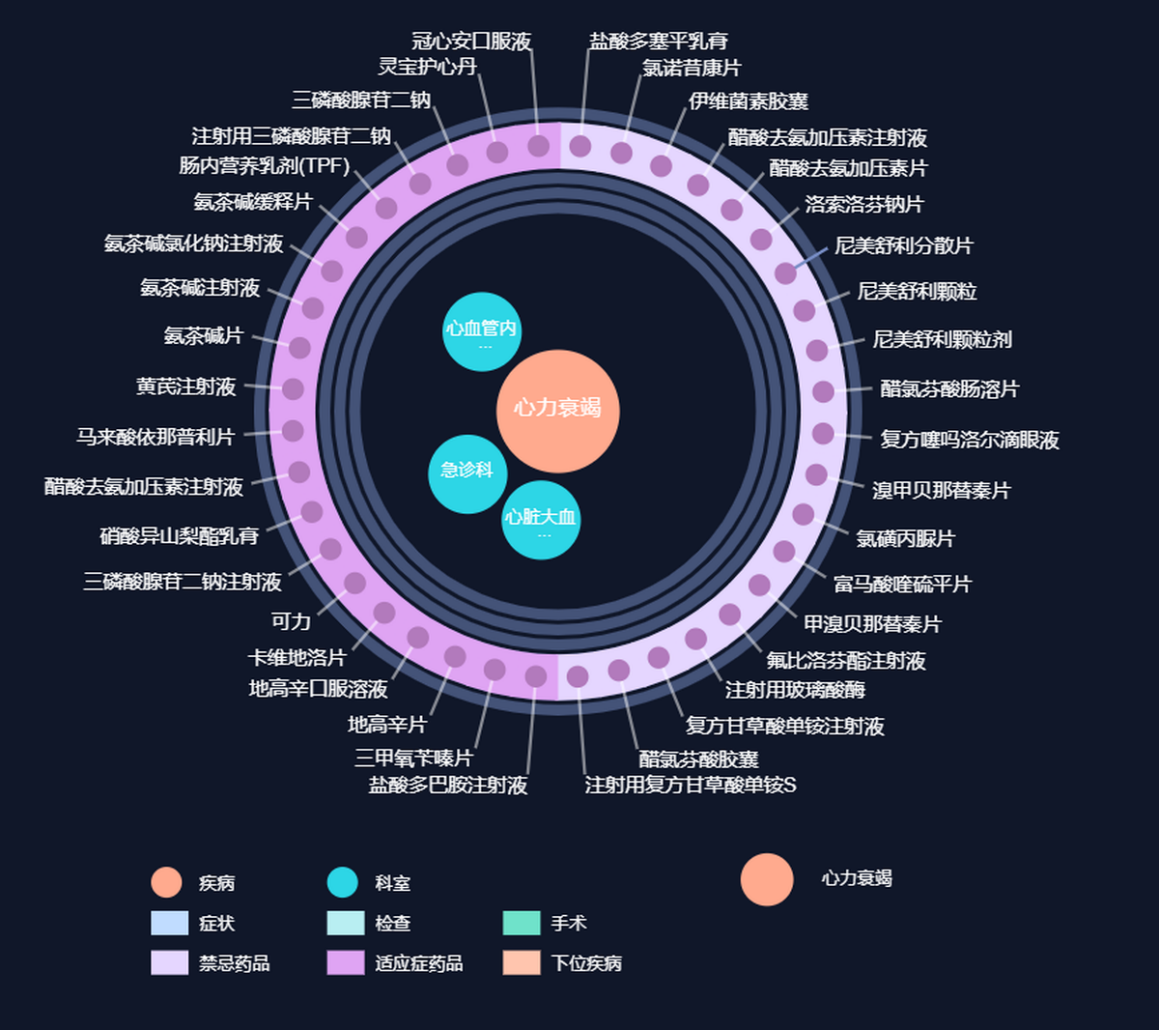
Snapshots of our knowledge graph data.

Finally, we built a medical knowledge graph that includes 1,616,549 nodes and 5,963,444 edges. To make it easier to explore the graph, we developed a web app to support the browsing on our medical knowledge graph on a website [36], which is open and free to access. Figure 11 shows a snapshot of our knowledge graph data. In brief, when the user selects a concept, the concept will be shown in the center of the circle. The concepts belonging to the same category that show certain relationships with the central concept will be placed on the same ring, whereas different types of relations will have different colors for concepts on the same ring. For example, as shown in Figure 11, the node “心力衰竭 (heart failure)” is in the center. All drugs that are related to heart failure are on the same ring. The drugs having indication relations are in dark purple while those having contraindication relations are in light purple.

### FWA Detection in Claim Processing

#### Dataset

**Figure 12:**
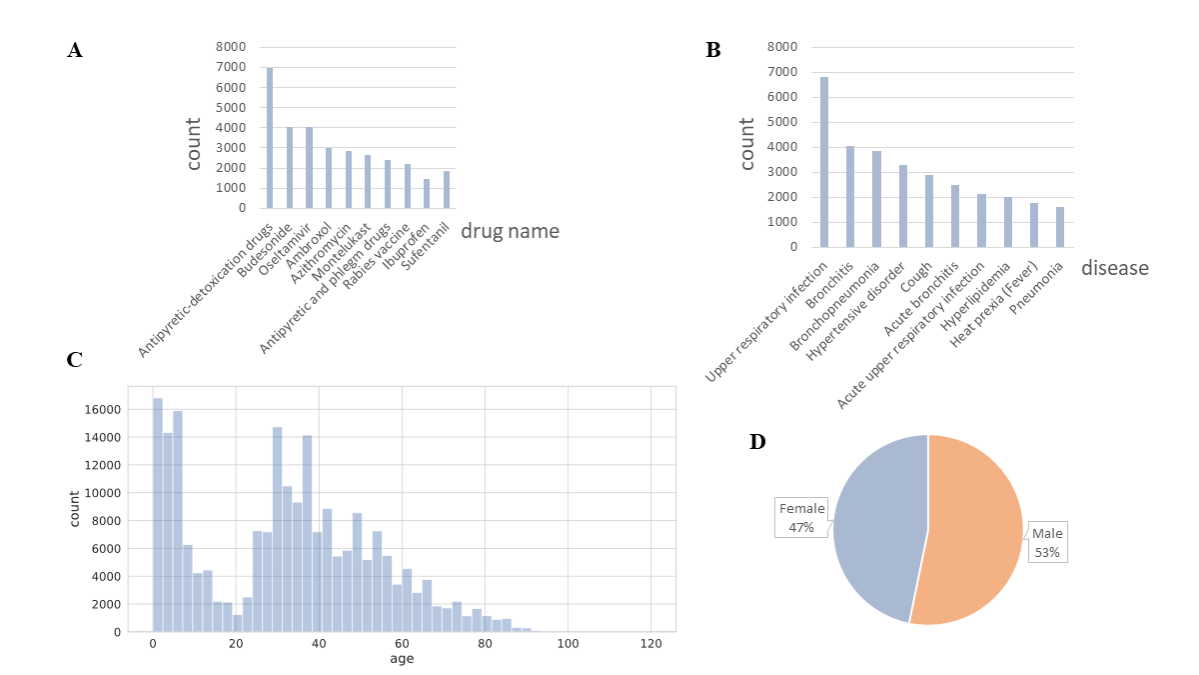
Statistical overview of claim documents: (A) top 10 drug names in claim documents; (B) top 10 diseases occurring in claim documents; (C) age distribution in claim documents; (D) sex ratio in claim documents.

We collaborated with the insurance company in our PingAn group and obtained 214,107 claim documents. Every claim document contains a list of diagnoses (1.5 diagnoses on average) and a list of drugs (2.3 drugs on average). There are 2586 unique ICD-10 codes and 5307 unique common drug names in these claim documents. More information is shown in Figure 12.

In the following, we report the performance of each step in the FWA detection process.

#### Subtask: Terminology Standardizing

As described above, the first step in FWA detection is to link the diagnosis and medications to the entities in our medical knowledge graph. Our proposed multilevel string matching algorithm ZhFuzzyED achieved 0.861 accuracy in linking the diagnosis to the ICD-10 coding system and 0.902 accuracy for drug normalization.

#### Subtask: Graph Reasoning–Based Relation Detection

For claim processing, 10% of claims are typically rejected for various reasons. The clinical unreasonable problem is only one of the reasons for rejection. We randomly selected 100 rejected claim documents and let the insurance inspector manually label the type of the rejected reasons. We then applied our proposed FWA detection method to identify the three types of frauds as described above. Table 9 lists the number of events that were labeled by humans and the number of events that were detected by the medical knowledge database–based method. Specifically, excess prescription means the drug has been abused in a document, fraud diagnosis reflected that there is no drug suite for the diagnosis, and irrational prescription is when a conflict exists in the drug list.

Our method could help detect around 70% of these events. This result is much better than the existing method that relies on only a human to check part of the claims randomly. Therefore, the existing method requires investing many professionals and spending a substantial amount of time to check each claim one by one.

**Table 9:** Performance of claim processing.

Subtask	Events (n)	Detected by the MKG^a^ (n)	Graph reasoning rule (Cypher-like syntax)	Claim example
Excess prescription	7	5	(Disease)-[:is_a]-(Disease)<-[:indication]-(Drug)-[:is_a*0..1]->(ATC)<-[:is_a*0..1]-(Drug)	Diagnosis: Acute bronchitis Drug: Bifidobacterium double live bacteria powder (*excess prescription*)
Fraud diagnosis	11	7	(Drug)-[:is_a]->(ATC)<-[:is_a]-(Drug)-[:indication]->(Disease)-[:is_a]-(Disease)	Diagnosis: Guillain-Barré syndrome, *Thyroid nodules (fraud diagnosis)* Drug: Immunoglobulin injection
Irrational prescription	4	3	(Drug)-[:is_a]->(ATC)-[:is_a*0..3]->(ATC)<-[:is_a*0..3]-(ATC)<-[:is_a]-(Drug)	Drug: *Atorvastatin calcium tablets, Ketoconazole cream (there is an interaction between the two drugs)*

^a^MKG: medical knowledge graph.

## Discussion

### Principal Results

In this paper, we have proposed an automatic method to extract information from medical knowledge to build a medical knowledge graph specifically for FWA detection. First, our NER results showed that by integrating the hand-crafted features with the embeddings helps to improve the accuracy of medical entity recognition. In addition, when the domain-specific rules were added, the performance could be further improved as shown in Table 7.

Second, for medical relation extraction, the PCNN+AT model showed better performance as compared to CNN or PCNN. Third, we constructed a high-quality medical knowledge graph, including 1,616,549 nodes and 5,963,444 edges. Finally, we designed the rules on top of the medical knowledge graph to detect three kinds of FWAs in claim processing. The experimental results showed that our approach helps to detect 70% of these FWA events automatically. The medical knowledge graph–based method provided good interpretability of the results. The reasoning process on the medical knowledge graph can help the insurance inspector to quickly determine whether the claim should be rejected, which will contribute to substantial savings in the claim processing cost. Our system has already been deployed as a service to generate alerts of suspected claims for insurance inspectors within our PingAn group.

### Limitations

Our medical knowledge graph and proposed rules could detect three kinds of FWA issues. However, there are still other types of FWA events such as medication overdose and medications that are not suitable for the population. Therefore, we need to integrate more information into our medical knowledge graph and design more rules to detect more types of FWA problems. In addition, our method still missed some FWA events. This is because we failed to extract some drug categories from the drug labels. Therefore, we need to further improve the recall of our information extraction method.

### Conclusions

In this study, we examined the effectiveness of building a medical knowledge graph to enhance FWA detection on claim data. Our method can help insurance inspectors to identify insurance claims worthy of attention from thousands of documents and ultimately reduce Medicare and Medicaid spending.

## Abbreviations

AT: adversarial training
ATC: Anatomical Therapeutic Classification
BERT: Bidirectional Encoder Representations from Transformations
BiLSTM: bidirectional long short-term memory
BIO: Beginning-Inside-Outside
CNN: convolutional neural network
CRF: conditional random field
DDI: drug-drug interaction
FWA: Fraud, Waste, and Abuse
ICD: International Classification of Diseases
NER: named entity recognition
PCNN: piecewise convolutional neural networks
